# Use of a toll-free call center for COVID-19 response and continuity of essential services during the lockdown, Greater Kampala, Uganda, 2020: a descriptive study

**DOI:** 10.11604/pamj.2024.47.141.36203

**Published:** 2024-03-26

**Authors:** Elizabeth Katana, Alex Ndyabakira, Richard Migisha, Doreen Nsiimire Gonahasa, Geoffrey Amanya, Aggrey Byaruhanga, Isaiah Chebrot, Christopher Oundo, Daniel Kadobera, Lilian Bulage, Alex Riolexus Ario, Daniel Ayen Okello, Julie Rebecca Harris

**Affiliations:** 1 Uganda Public Health Fellowship Program, Ministry of Health, Kampala, Uganda,; 2 Directorate of Public Health and Environment, Kampala Capital City Authority, Kampala Uganda,; 3 Uganda National Institute of Public Health, Ministry of Health, Kampala, Uganda,; 4 Division of Global Health Protection, US Centers for Disease Control and Prevention, Kampala, Uganda

**Keywords:** Toll-free, call center, capital city, COVID-19 response, social services, access, continuity, lockdown, Uganda

## Abstract

**Introduction:**

on March 21, 2020, the first case of COVID-19 was confirmed in Uganda. A total lockdown was initiated on March 30 which was gradually lifted May 5-June 30. On March 25, a toll-free call center was organized at the Kampala Capital City Authority to respond to public concerns about COVID-19 and the lockdown. We documented the set-up and use of the call center and analyzed key concerns raised by the public.

**Methods:**

two hotlines were established and disseminated through media platforms in Greater Kampala. The call center was open 24 hours a day and 7 days a week. We abstracted data on incoming calls from March 25 to June 30, 2020. We summarized call data into categories and conducted descriptive analyses of public concerns raised during the lockdown.

**Results:**

among 10,167 calls, two-thirds (6,578; 64.7%) involved access to health services, 1,565 (15.4%) were about social services, and 1,375 (13.5%) involved COVID-19-related issues. Approximately one-third (2,152; 32.7%) of calls about access to health services were requests for ambulances for patients with non-COVID-19-related emergencies. About three-quarters of calls about social services were requests for food and relief items (1,184; 75.7%). Half of the calls about COVID-19 (730; 53.1%) sought disease-related information.

**Conclusion:**

the toll-free call center was used by the public during the COVID-19 lockdown in Kampala. Callers were more concerned about access to essential health services, non-related to COVID-19 disease. It is important to plan for continuity of essential services before a public health emergency-related lockdown.

## Introduction

During pandemics, the World Health Organization (WHO) recommends that multiple avenues be used to carry out effective risk communication for the public [1]. One component of risk communication is providing ways for the public to obtain answers to relevant questions. This can be especially important during an outbreak of a new disease such as COVID-19 that may be subject to new and rapidly evolving information [2].

One approach to effective risk communication involves the use of call centers for the public. The stand-up of call centers during public health emergencies can divert unnecessary calls from the usual emergency telephone systems and provide relevant information to the public during the response [3]. Examples of outbreaks where the use of call centers has been documented include the 2004 Severe Acute Respiratory Syndrome (SARS) outbreak in Hong Kong and Singapore [4,5], the 2009 swine flu pandemic in China [6], and the 2014 West Africa Ebola outbreak in Sierra Leone and Guinea [7,8]. While the Ugandan public can call a national emergency toll-free line through the Uganda Police or utilize private ambulances and community support networks during normal times [9], the COVID-19 epidemic called for dedicated measures to address the concerns of the public.

On March 21, 2020, the first case of COVID-19 was confirmed in Uganda [10] and the country entered a total lockdown on March 30 [11]. The total lockdown comprised a nationwide curfew from 7: 00 pm to 8: 00 am and a ban on both public and private motor vehicle movement. Essential services, including access to health facilities and movement of food trucks or cargo, were exempted and the government began food and relief item distribution. Excess government vehicles were availed for use by the districts to support the continuity of essential health services through the provision of transportation to health facilities, with priority given to medical emergencies, hospitalization, referrals, prenatal care, childbirth, and neonatal care [12]. The lockdown was gradually lifted from May 5 - June 3, starting with allowing some essential businesses to reopen, allowing private vehicles to move, and reopening retail shops, hotels, and restaurants with standard operating procedures (SOPs) in place. From June 4 - June 30, the final phase of reopening included the return of public transport, allowing additional passengers in vehicles, and reopening of the remaining businesses [13].

On March 25, 2020, an emergency operations center (EOC) with a toll-free call center was set up at the Kampala Capital City Authority (KCCA). A trained team of call attendants, clinicians, and epidemiologists operated the call center in collaboration with rapid responder teams, ambulance drivers, and drivers for community vehicles. The call center was intended to primarily address COVID-19 health-related concerns and allow the public to get information related to COVID-19 or access to essential services including transport to health facilities, food, water, and sanitation (WASH) issues, and other general inquiries [14].

Compared with other countries, Uganda experienced a very slow start to its COVID-19 epidemic [15]. However, the call center was still utilized by the public. We documented the setup and use of the call center and analyzed key concerns raised by the public during the COVID-19 lockdown in Greater Kampala. We also documented important lessons learned to inform policy and make recommendations for the implementation of future similar projects.

## Methods

**Study setting and design:** Kampala Capital City Authority is the administrative center for Greater Kampala and is a semi-autonomous body governing on behalf of the Ugandan central government. Kampala is the capital of and largest city in Uganda with an estimated population of approximately 1.7 million people. It is among the fastest-growing cities in Africa [16,17]. The private health sector dominates Kampala´s health system which has close to 1,500 private facilities and 106 public health facilities [18]. Kampala is mostly encircled by Wakiso District, which has an additional 2 million people and forms a large part of the Greater Kampala Metropolitan Area (GKMA) [19]. The rest of the GKMA comprises the nearby urban areas of Mukono, Mpigi, Buikwe, and Luweero districts.

On April 11, 2020, a government exercise to distribute food and relief items to persons unable to work during lockdown was rolled out in GKMA. By May 10, 2020, it was reported that nearly half of the suburbs in greater Kampala had not yet received any relief food [20]. This exercise concluded on May 28, 2020, amid significant criticism that much of the public had not benefitted from the food distribution [21]. During this descriptive assessment period for the call center (March 25 to June 30, 2020), the country had 889 COVID-19 confirmed cases, 184 active cases as of June 30, and 0 deaths due to COVID-19, and some of the earlier restrictions including bans on public and private transport had been eased [22].

**Setting up the COVID-19 toll-free call center:** the COVID-19 toll-free call center was set up at the EOC of the Directorate of Public Health and Environment at the Kampala Capital City Authority (KCCA) on March 25, 2020, and was in operation until June 30, 2020. Two call center hotlines were established by the KCCA; the numbers were disseminated to the public through all mass media outlets in the GKMA, press briefings and releases, KCCA and MOH official websites, and social media platforms including WhatsApp, Twitter, and Facebook. The toll-free call center was open every day, 24 hours a day. It was directly manned by a team of 8 call attendants, 4 clinicians, 2 epidemiologists, 2 information technology (IT) specialists, and 4 supervisors from the Directorate of Public Health and Environment. All were volunteers with a minimum of a Uganda Advanced Certificate of Education. At any one time, the lines were manned by at least two call attendants and at least one of each of the other cadres. The team was trained in basic effective communication, health facility referral and ambulance systems, COVID-19 community case definition, and key surveillance components. In addition to regular training, the team was provided with general office supplies and equipment including computers, headsets and microphones, software to maintain call records, and telephone contacts of district leaders, security staff, and relief food distribution staff.

**Figure 1:**
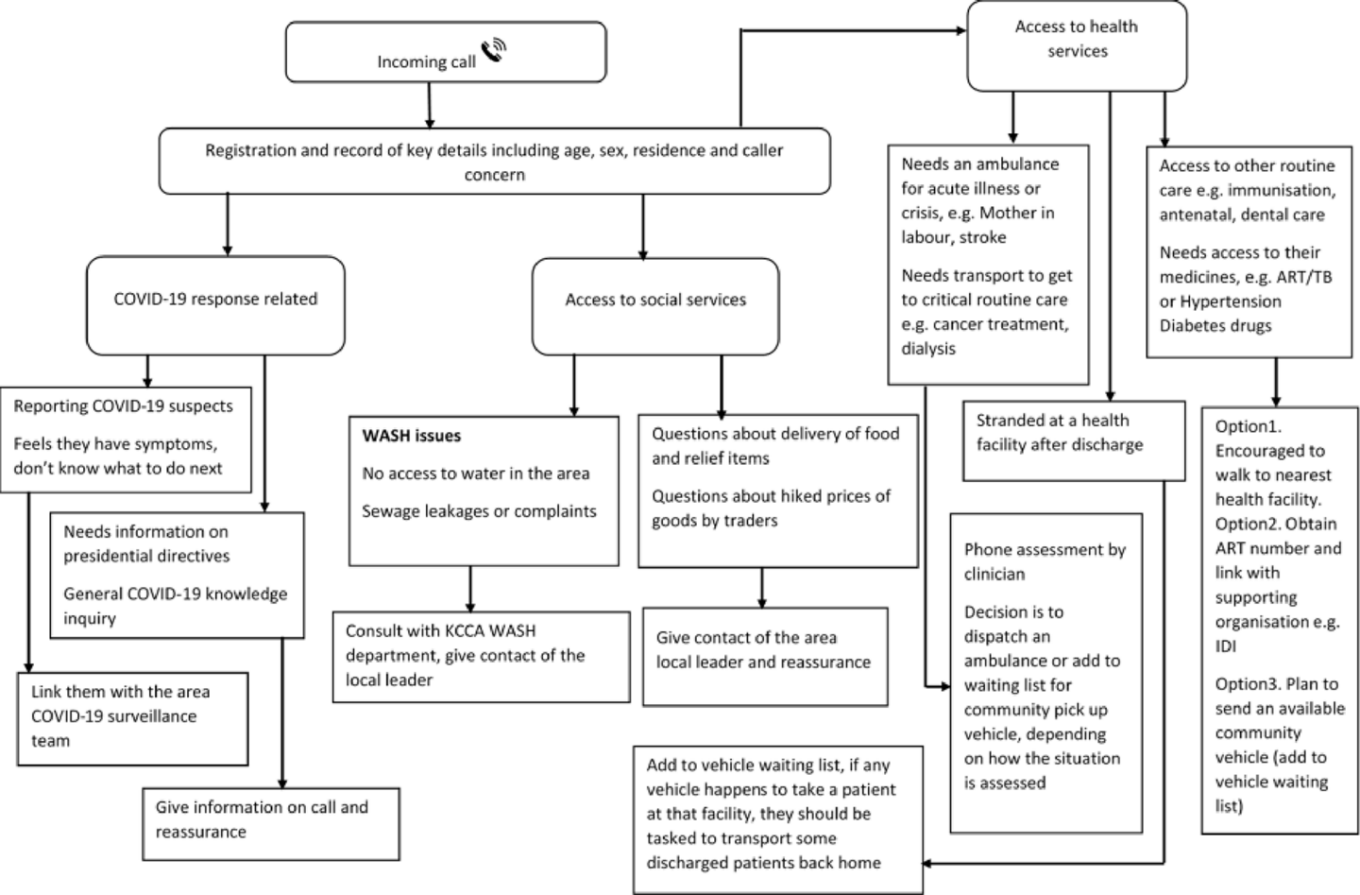
flow chart illustrating the telephone triage process used at a toll-free call center for COVID-19 response and continuity of essential services during the lockdown in Greater Kampala, Uganda, 2020

The EOC response team comprised evacuation teams with ambulance and community vehicle drivers who were trained in the evacuation of patients and rapid response. These were dispatched directly by call attendants following clear rapid response team dispatch protocols. A total of 14 community pickup vehicles and 11 ambulances were deployed and stationed along the four major GKMA highways and at KCCA public health facilities. Patients were transported to the nearest health facility including private facilities on their route as determined by the clinician on call. Those who needed specialized services only available at referral levels facilities including cancer care, dialysis, and elective surgeries were transported directly to the applicable facility. Standard operating procedures for the telephone triage process were developed based on U.S. Centers for Disease Control guidance for setting up call centers during public health emergencies [23]. The call center team assessed the caller´s concerns during the initial call and provided directives based on the urgency. The call attendant consulted the EOC clinician or epidemiologist as needed (Figure 1).

**Study variables:** we categorized data on the incoming calls into four domains including COVID-19-related, health services, social services, and other inquiries. Demographic information including name, sex, residence, telephone contact, date, and time of call was recorded. COVID-19-related domain included categorization for those reporting suspected cases, requiring general disease information or clarification on the presidential directives. Health services included ambulance requests for acute illnesses and emergencies (including COVID-19), requests for transportation to access routine care, or persons stranded at health facilities during lockdown. Social services included WASH, concerns about price hikes for goods, and requests for food or other relief items. Other concerns included legal and revenue collection issues, security concerns, and job seekers. These categorizations were determined through observations of patterns of caller concerns during the data collection process.

**Data management:** from March 25 to April 13, 2020, call data were directly entered into an MS Excel 2019 database designed by epidemiologists on the team. On April 14, 2020, the call center adopted an online electronic questionnaire developed by the team, which was based on the initial questionnaire. Epidemiologists cross-checked all collected primary data daily from questionnaires for completeness, correctness, and consistency. All data were saved and backed up regularly in a password-protected computer. There was restricted access to the call center's electronic systems, landlines, and personal data following Uganda´s Data Protection and Privacy Regulations [24].

**Figure 2:**
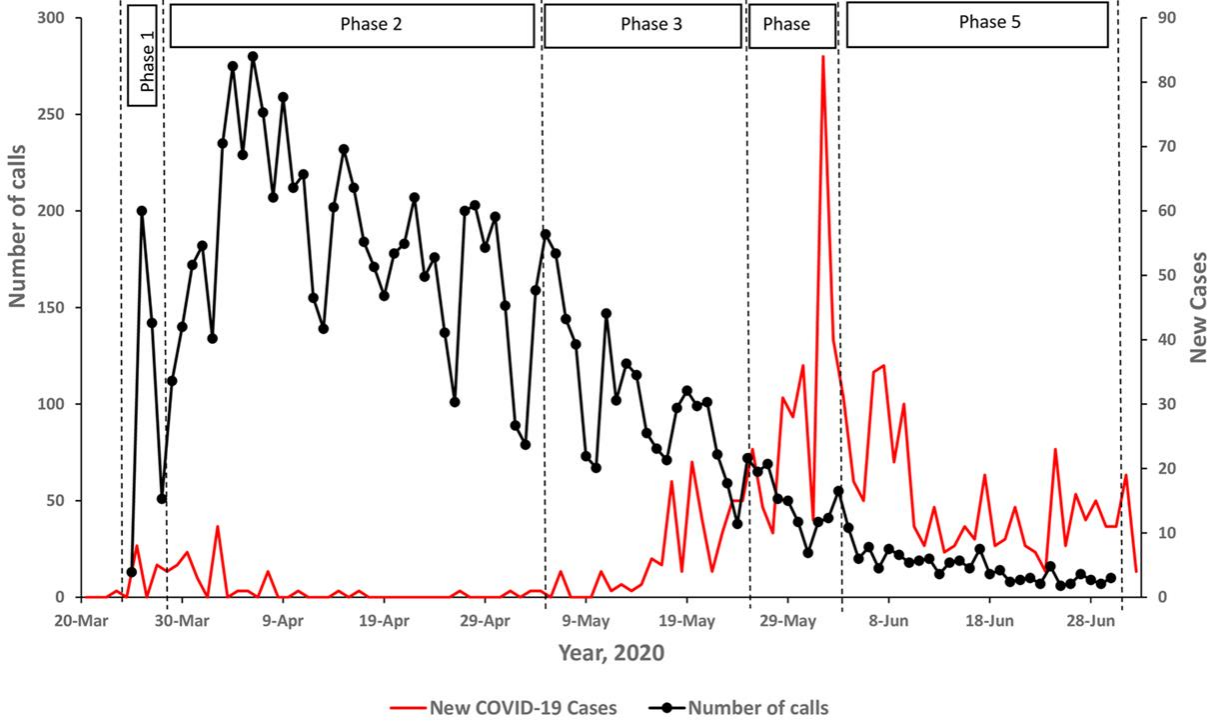
time trend of daily incoming calls versus new confirmed COVID-19 cases at the toll-free call center for COVID-19 response and continuity of essential services during five lockdown phases in Greater Kampala

**Data analysis:** for the analysis, the lockdown directives and easing of the restrictions due to the COVID-19 outbreak were categorized into five phases (Figure 2). “Partial lockdown” was instituted on March 25 - March 29 and included a ban on public transport. “Total lockdown” on Mar 30 involved a ban on private cars and a nationwide curfew from 7 pm to 6: 30 am until May 5. The government exercise to distribute food and relief items to vulnerable persons commenced on Apr 11 and was concluded on May 28. “Partial lifting phase 1” allowed some essential businesses to operate from May 5 to May 25. “Partial lifting phase 2” allowed private vehicles to move, and more shops, hotels, and restaurants with standard operating procedures (SOPs) to operate from May 26-June 3. “Partial lifting phase 3” began on June 4 - June 30 and included the return of public transport and one additional person in a private vehicle as well as more businesses to operate [25]. We summarized the call data into four domains. These included calls related to the COVID-19 response, requests to access health services (excluding those related to COVID-19), requests for assistance accessing social services, and other general inquiries. No caller observation had missing information on any of the domains. We conducted descriptive analyses of the public concerns during the different phases of the lockdown and easing of the restrictions using MS Excel 2019.

**Ethical consideration:** this activity was part of the public health emergency response for COVID-19 at the Directorate of Public Health and Environment (DPHE) at the Kampala Capital City Authority (KCCA), the governing body of Kampala city under the jurisdiction of the central government. This study was not reviewed or approved by any institutional review board (ethics committee) before the study began as it was part of the public health emergency response. The Uganda Ministry of Health (MOH) gave the directive to conduct epidemiological investigations regarding the COVID-19 epidemic response in the city. This directive applied to activities involving primary data collection. We sought permission to abstract and use the routinely generated toll-free call center data from the Directorate of Public Health and Environment at the Kampala Capital City Authority. This study used call center records, no participant enrollment was involved, and all the data were fully anonymized before we accessed them. Therefore, data on unique identifiers including names and telephone numbers were not retrieved during this assessment to protect the confidentiality of the callers. The abstracted data was stored on a password-protected computer and was not shared with anyone outside this investigation team. Additionally, The Office of the Associate Director for Science, U.S. Centers for Disease Control and Prevention, determined that this activity was in response to a public health emergency with the primary intent of informing public health practice (epidemic disease control activity). It was determined therefore to not be human subjects research as we received a project determination as non-research and clearance from the Centers for Disease Control and Prevention (CDC) to conduct this assessment.

## Results

**Description of incoming calls at a toll-free call center for COVID-19 response and continuity of essential services during the lockdown, Greater Kampala, Uganda, March - June 2020:** in total, there were 10,167 incoming calls answered and recorded during the 3 months of the evaluation. Of these, 62% were received during the total lockdown period, predominantly from the Kampala and Wakiso areas (91%). Among all calls, 65% involved issues with access to health services, 14% were about COVID-19-specific issues and 15% were about access to social services. An additional 6% were about other inquiries, such as legal and revenue collection issues (Table 1).

**Table 1:** summary of the incoming calls at a toll-free call center for COVID-19 response and continuity of essential services during five lockdown phases in Greater Kampala, Uganda, 2020

Parameter (n=10,167)	Number of calls	(%)
Lockdown phase		
1.Partial lockdown	830	(8.2)
2.Total lockdown	6,341	(62.4)
3. Partial lifting phase 1	2,147	(21.1)
4. Partial lifting phase 2	432	(4.2)
5. Partial lifting phase 3	417	(4.1)
Caller residence (region)		
Kampala	6,005	(59.1)
Wakiso	3,198	(31.5)
Mukono	336	(3.3)
Other central	369	(3.6)
Eastern	141	(1.4)
Northern	32	(0.3)
Western	85	(0.8)
Caller issues		
Access to health services	6,578	(64.7)
Other inquiries*	649	(6.4)
COVID-19-related	1,375	(13.5)
Access to social services	1,565	(15.4)

* Primarily included legal and revenue collection issues, security concerns, and job seekers

**Figure 3:**
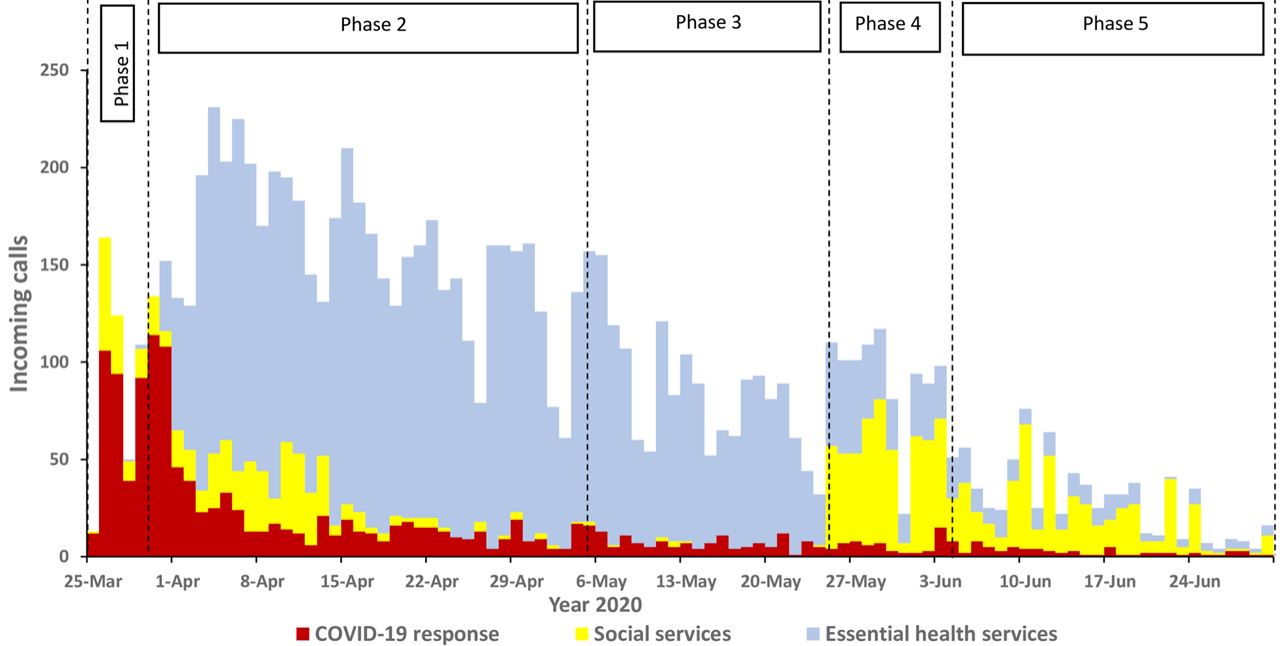
the trend of daily incoming calls about COVID-19, access to social services, and essential health services at the toll-free call center in Greater Kampala

**Figure 4:**
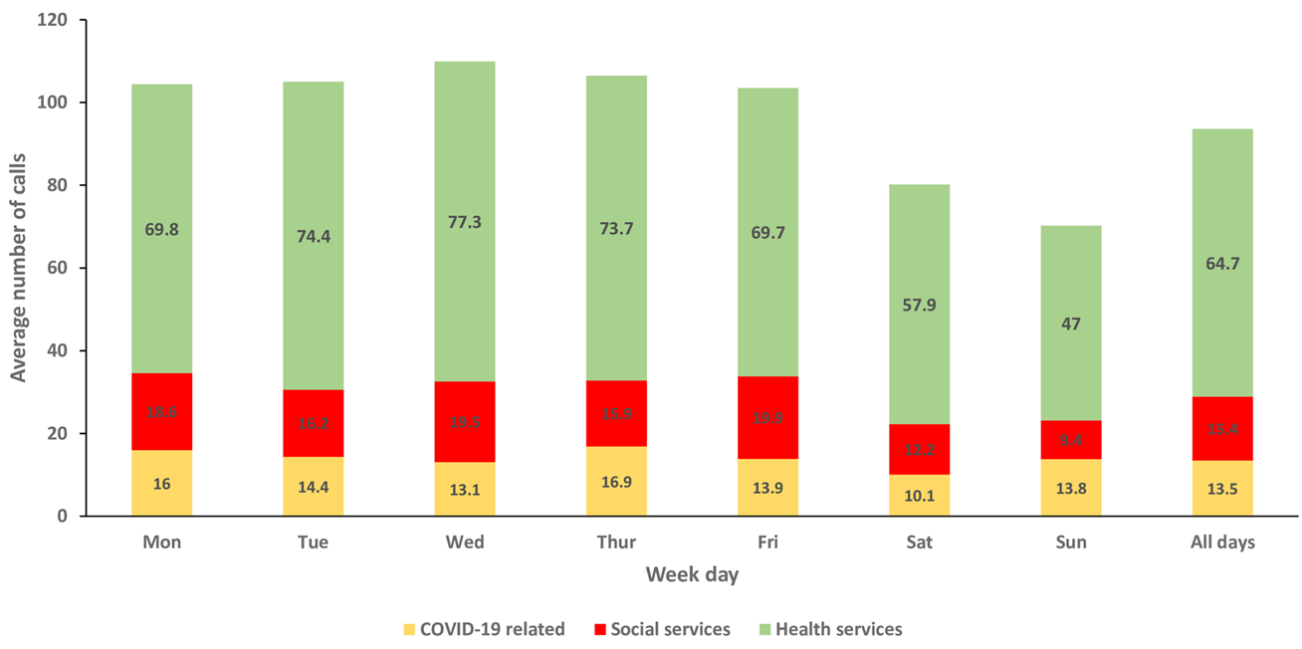
the average number of incoming calls, about COVID-19, access to social services, and essential health services, by day of the week, at the toll-free call center in Greater Kampala

Trend of daily incoming calls at the toll-free call center for COVID-19 response and continuity of essential services during the lockdown, Greater Kampala, Uganda, March - June 2020: on average, more than 100 calls were received daily during the total lockdown. The number of calls declined gradually as the lockdown was lifted (Figure 2). By topic area, calls concerning access to essential health services dominated throughout the entire study period. The number of calls requesting access to social services was high at the start of the total lockdown, gradually reduced throughout late April and mid-May, and suddenly increased during the partial lifting phase 2 in late May as the government program to distribute food and relief items in the communities was concluded on May 28. After an initial influx of calls about COVID-19 in the first few weeks, calls about COVID-19 comprised a small proportion of overall calls (Figure 3). More calls were received during weekdays as compared to weekend days. Calls concerning health services were predominant throughout the week (Figure 4).

Incoming calls per day for various reasons at a toll-free call center for COVID-19 response and continuity of essential services during the lockdown, Greater Kampala, Uganda, March - June 2020: one-third (33%) of the 6,578 calls about access to health services were requests for ambulances for patients with acute illnesses and medical emergencies (unrelated to COVID-19), 18% were from persons stranded at health facilities during the lockdown, and 15% were from mothers in labor. Other health services such as dog bites, domestic violence, dental issues, elective surgeries, and eye care contributed 17% of the calls for essential health services. In total, 76% of the 1,565 calls about social services were requests for food and relief items and 13% were about illegal price hikes from businesses, which the public was encouraged to call to report. Fifty-three percent of the 1,375 calls about COVID-19 response were seeking COVID-19 disease-related information and 26% were reporting COVID-19 suspected cases (Table 2). There were no calls about COVID-19 emergencies.

**Table 2:** average number of incoming calls per day for various reasons at a toll-free call center for COVID-19 response and continuity of essential services during the lockdown in Greater Kampala, Uganda, 2020

Category (N=10,167)	Frequency	(%)	Daily average	Standard deviation (SD)
Access to essential health services	6,578	(64.7)	67.1	(58.0)
Ambulance requests for emergencies and acute illnesses*	2,152	(32.7)	22.0	(17.5)
Stranded at hospitals after discharge	1,155	(17.6)	11.8	(12.8)
Other health services**	1,096	(16.7)	11.9	(11.2)
Mothers in labor due for delivery	1,004	(15.3)	10.2	(9.1)
Access to ART/TB drugs	430	(6.5)	4.4	(3.6)
Cancer treatment	283	(4.3)	2.9	(3.1)
Dialysis	140	(2.1)	1.4	(1.8)
Mental health issues	123	(1.9)	1.3	(1.6)
Routine immunization	96	(1.5)	1.0	(1.6)
Routine antenatal care	99	(1.5)	1.0	(4.8)
Access to social services	1,565	(15.4)	15.9	(19.2)
Requests for food/other relief items	1,184	(75.7)	12.1	(9.4)
WASH concerns	223	(14.2)	2.3	(2.7)
Reports of illegal price hikes	158	(10.1)	1.6	(7.0)
COVID-19 response	1,375	(13.5)	14.0	(22.5)
Seeking disease-related information	730	(53.1)	7.4	(10.9)
Reporting suspected cases	360	(26.2)	3.7	(16.4)
Seeking clarification on presidential directives	285	(20.7)	2.9	(6.2)
Other inquiries***	649	(6.4)	(6.6)	(4.8)

* Primarily included heart issues/stroke, respiratory distress, sickle cell crisis, asthma attacks, and road accidents; **primarily included dog bites, domestic violence, dental issues, elective surgeries, and eye care; *** primarily included legal and revenue collection issues, security concerns, and job seekers. ART = antiretroviral treatment; TB = tuberculosis; WASH = water sanitation and hygiene

## Discussion

The establishment of an emergency call center during the start of the COVID-19 pandemic in Uganda and the associated national lockdown revealed key community concerns associated with both COVID-19 and the lockdown. A high volume of calls was received at the call center, suggesting that the call center was needed by the Ugandan public. Most calls were related to continuity of essential health services, including emergencies and acute illnesses not directly related to COVID-19, with the most pressing concerns being about accessing transportation to and from health facilities during lockdown. Beyond providing key information to the public, some of the data collected from the call centers might be used to assess the effectiveness of existing public health and social support strategies. The Ugandan government implemented locally-based strategies to ensure the continuity of essential services during the COVID-19 lockdown. For example, the public could contact local leaders to seek written permission and clearance to move sick persons and to access social services, including food. In addition, the government established a program for the distribution of food and other relief items in the communities [12]. Despite these efforts, as the relief food distribution concluded in late May, calls about the lack of receipt of relief food increased. These calls suggested that the food distribution might not have been implemented as completely as desired by the public. In addition, many Ugandans reported being unable to reach their local leaders for permission to move the sick. Others reported that they could not access private vehicles or ambulances even after receiving clearance, and were forced to walk to health facilities [26]. While the call center was able to link callers with ambulances and provide contact information for local leaders, issue resolution was not tracked, nor was a customer satisfaction survey implemented. These should both be considered as a monitoring metric for future similar interventions, to continue to improve call center implementation and as a way to measure effectiveness.

This was the first time a nationwide lockdown and curfew were instituted in response to a public health emergency in Uganda. A lockdown is considered an extreme public health measure that can bring about public panic, requiring effective communication [27]. Studies have shown that people´s awareness and social responses will evolve simultaneously as an epidemic evolves [28]. Considerably fewer calls were received in all three lockdown lifting phases compared with the total lockdown phase. The high number of early calls is likely related to both initial public anxiety during the total lockdown and curfew, and fears about a rapid expansion of COVID-19 cases similar to what was being experienced elsewhere. However, the growth of the COVID-19 outbreak in Uganda at that time was extremely slow [29]. As the feared increase in cases failed to materialize, the focus of the calls shifted to mitigating the ancillary effects of the lockdown. In contrast, COVID-19-related call centers in Europe, where the outbreak was expanding exponentially, faced consistently higher volumes of calls [30]. The findings of our assessment highlight the importance of the call center in disseminating information and communicating with the public during the COVID-19 response. Although the center was set up primarily to address COVID-19 health-related concerns and allow the public to get information related to COVID-19 or access to essential services, it was used primarily for the latter. A call center can be an integral element of response during an epidemic-related lockdown. We recommend further evaluation of the effectiveness of the call center in addressing some of the public concerns during an epidemic crisis.

The study focused on documenting the public concerns directed toward the call center. However, there was limited tracking of issue resolution and as a result, we were not able to explore the outcomes after the phone calls, such as eventual access to health services or food and other relief items. Because of this, the effectiveness of the call center in addressing issues is still unknown. Additionally, data on unanswered calls or outgoing calls were not collected, and therefore we were unable to illustrate the total call volume and community need for the call center. Despite the limitations, our study highlights the role played by the call center in the COVID-19 response.

## Conclusion

The toll-free call center was utilized by the public to obtain information on the COVID-19 response and make inquiries on health and social services during the lockdown. The call center was more utilized in providing information on access to essential health services compared to the primarily intended COVID-19 response. A call center for an epidemic response can facilitate other actions or developments and should be an integral element, during an epidemic-related lockdown.

### What is known about this topic

- *Call centers have been credited as a successful approach to achieving effective risk communication for the public during the response to public health emergencies, especially during an outbreak of a new disease such as COVID-19 that may be subject to new and rapidly evolving information;*
- *Call centers can divert unnecessary calls from the usual emergency telephone systems and provide relevant information to the public during the emergency response and have been used in outbreak responses in the past including the 2004 Severe Acute Respiratory Syndrome (SARS) outbreak in Hong Kong and Singapore, the 2009 H1N1 flu pandemic in China, and the 2014 West Africa Ebola outbreak in Sierra Leone and Guinea;*
- *However, there have been no assessments done to evaluate the setup and use of toll-free call centers during large epidemics including COVID-19 in the Ugandan setting*.

### What this study adds

- *This study highlights that the call center was used by the public in Greater Kampala to obtain information and they were more concerned about access to essential health services, during the COVID-19 lockdown as opposed to COVID-19-related issues;*
- *Call centers can support other actions or developments during response to large outbreaks and should be implemented alongside user satisfaction surveys to help understand how they can be effective in addressing these issues;*
- *Additionally, during future similar epidemics, implementation of interventions such as lockdowns should be focused on ensuring access to essential health services*.
